# Formation of Mercury(II)-Glutathione Conjugates Examined Using High Mass Accuracy Mass Spectrometry

**DOI:** 10.4236/ijamsc.2013.12011

**Published:** 2013-12

**Authors:** Zachary Fine, Troy D. Wood

**Affiliations:** 1 Department of Chemical and Biological Engineering, University at Buffalo, State University of New York, Buffalo, USA; 2 Department of Chemistry, University at Buffalo, State University of New York, Buffalo, USA

**Keywords:** Glutathione, Mercury, FT-ICR, Mass Spectrometry, Tandem Mass Spectrometry

## Abstract

Maternal exposure to Hg(II) during pregnancy has been identified as a potential causal factor in the development of severe neurobehavioral disorders. Children with autism have been identified with lower reduced glutathione (GSH)/oxidized glutathione (GSSG) ratios, and GSH is known to strongly bind Hg(II). In order to gain insight into the mechanism by which GSH binds Hg(II), high resolution mass spectrometry coupled with tandem mass spectrometry was utilized to examine the conjugation process. While the 1:1 Hg(II):GSH conjugate is not formed immediately upon mixing aqueous solutions of Hg(II) and GSH, two species containing Hg(II) are observed: the 1:2 Hg(II):GSH conjugate, [(GS)_2_Hg + H^+^], and a second Hg(II)-containing species around *m/z* 544. Interestingly, this species at *m/z* 544 decreases in time while the presence of the 1:1 Hg(II):GSH conjugate increases, suggesting that *m/z* 544 is an intermediate in the formation of the 1:1 conjugate. Experiments using the high mass accuracy capability of Fourier transform ion cyclotron resonance (FT-ICR) mass spectrometry coupled to an electrospray ionization source indicate that the intermediate species is [GSH + HgCl]^+^, and not the 1:1 conjugate [Hg(GSH) − H + 2H_2_O]^+^ postulated in previous literature. Further confirmation of [GSH + HgCl]^+^ is supported by collision of induced dissociation experiments, which show neutral loss of HCl from the intermediate and loss of the N- and C-terminal amino acids, indicating binding of Hg(II) at the Cys residue.

## 1. Introduction

Glutathione (GSH, *γ*Glu-Cys-Gly) is a tripeptide with a reactive thiol group found in relatively high intracellular concentrations, and it is the primary regulator of cellular redox homeostasis paired with its disulfide (GSSG) [1,2]. Some heavy metals such as Hg(II) are toxic to cells because of their ability to deplete GSH [3]. The binding constant of Hg(II) to GSH is very large as measured by polarographic [4] and nuclear magnetic resonance [5] methods. Zalups has expertly reviewed the role of thiol-containing proteins, including GSH, and their interactions with Hg(II) in uptake, accumulation, transport, and toxicity [6].

Electrospray ionization mass spectrometry (ESI-MS) has been used previously to examine the conjugation of Hg(II) with GSH [7–11]. The studies of Rubino *et al*. provided insight into the stoichiometry of binding between Hg(II) and GSH, with particular attention on the dissociation behavior of the conjugates under collisionally induced dissociation (CID) conditions by tandem mass spectrometry (MS/MS) [9,11]. Interestingly, it was found that the 1:1 Hg(II):GSH conjugates require significantly higher collisional activation energy than the 1:2 Hg(II):GSH or the 1:1 Hg(II):GSSG conjugates, suggesting strong coordination of Hg(II) at the carboxy terminus of GSH [9]. Burford *et al*. examined Hg(II) conjugation to GSH in both positive ion and negative ion ESI-MS, and also found the existence of 1:1 and 1:2 Hg(II):GSH conjugates [10]. Burford *et al*.’s results were replicated by positive ion ESI-MS and it was found that Hg(II):GSH conjugates spiked into plant extracts could be recovered and detected [8]. Of particular note was the publication of detailed isotopic insets, which proved that Hg(II) was indeed conjugated to GSH [8]. A mercury-glutathione conjugate with 1:3 Hg(II):GSH stoichiometry has been shown in negative ion mode ESI-MS [7].

Here, we report on the conjugation between Hg(II) and GSH using high resolution mass spectrometry. Our motivation for this investigation stems from three items. First, lower GSH:GSSG ratios in the plasma of children with autism have been found, which have been related to oxidative stress [12]. Second, as detailed thoroughly in reviews [13,14], there is an extensive evidence that exposure to mercury leads to neurological conditions. Third, all the previous ESI-MS reports were done on relatively low-resolution mass spectrometers, and we felt that an investigation using high resolution mass spectrometry would be useful to validate earlier studies and resolve existing ambiguities in interpretation of the ESI mass spectra from Hg(II):GSH conjugates. ESI coupled to Fourier transform ion cyclotron resonance mass spectrometry (ESI-FTICR) is used in the current investigation. The high resolution mass spectrometry data provide a keen insight into the mechanism by which Hg(II):GSH conjugates form.

## 2. Experimental

All high resolution mass measurements were acquired using a Bruker Daltonics (Billerica, MA) 12 tesla SolariX FT-ICR mass spectrometer equipped with an ESI source. Mercuric nitrate (J. T. Baker, Phillipsburg, NJ) and reduced glutathione (Sigma-Aldrich, St. Louis, MO) were prepared as separate aqueous solutions, each at 10 μg/mL. These were then mixed in a 1:1 volume:volume ratio and then loaded into a syringe for infusion into the ESI source. ESI was performed at 5.4 kV using N_2_ nebulizer gas (2.9 L/min) by infusing the sample mixture via syringe pump at 3.0 μL/min and applying N_2_ drying gas (2.8 L/min, 200°C) to desolvate the droplets. Ions were accumulated in the external quadrupole for 100 ms before transfer through the external ion optics to the FT-ICR trap. Ions were trapped in an Infinity cell (front trap plate 0.6 V, back trap plate 0.8 V) [15] operating at ~1 × 10^−9^ mbar, and excited and detected in broadband mode as positive ions over *m/z* 98.3-3000 using 2 MB time domain data sets; the data was zero-filled once and displayed in the magnitude mode. The resulting FT-ICR mass spectra are from the summation of 100 individual scans. Collision induced dissociation (CID) experiments were performed by isolation of the isotopic cluster in the mass-selective quadrupole and dissociation in the collision cell with argon gas (~1 × 10^−3^ mbar). All isotope distributions were simulated using the proposed ionic formulas using the IsotopePattern utility in Bruker Daltonics’ Compass software package.

## 3. Results and Discussion

**Figure 1:**
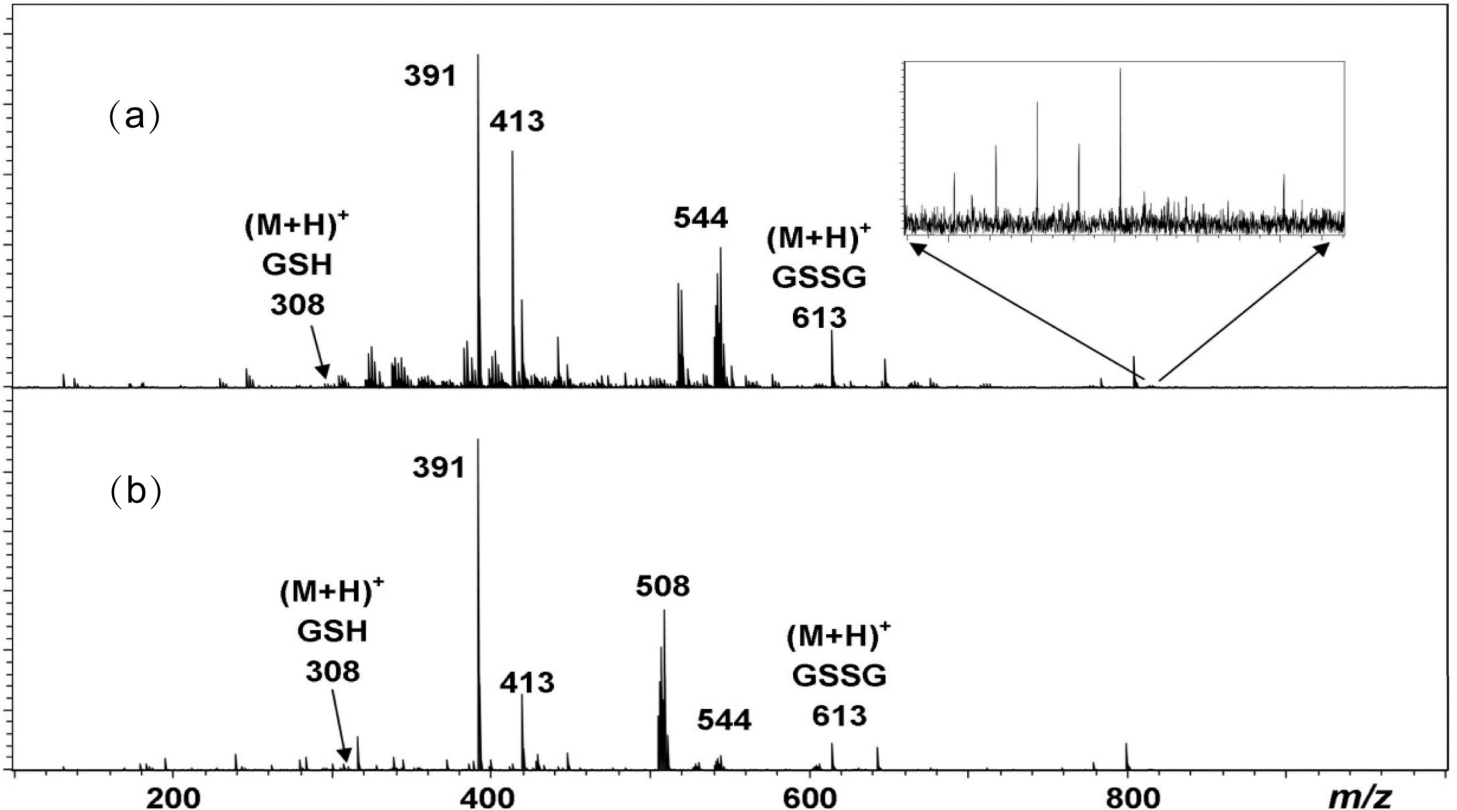
ESI-FTICR mass spectra of aqueous solutions of Hg(II) mixed with GSH (a) immediately after mixing (t = 0) and (b) after 4 days incubation at room temperature. The inset in Figure 1(a) represents the magnified region of *m/z* 810 – 820, indicating the presence [(GS)_2_Hg + H^+^], which is confirmed by accurate mass analysis.

Upon mixing 10 μg/mL aqueous solutions of mercuric nitrate and GSH, ESI-FTICR mass spectra were collected immediately and the resulting data is shown in Figure 1(a). One of the primary analytical advantages of FT-ICR is its ability to measure ionic masses with unparalleled accuracy [16]. Accurate mass measurements confirm the presence of (M + H)^+^ for both reduced glutathione (308.09317 Da, +2.1 mDa error) and oxidized glutathione (613.1633 Da, +4.1 mDa error). In addition, two peaks due to dioctyl phthalate, a common plasticizer, are observed, which include (M + H)^+^ at 391.2870 Da (+2.7 mDa error) and (M + Na)^+^ at 413.2690 Da (+2.8 mDa error) which serve as internal mass calibrants. As shown in the inset of Figure 1(a), a very weak peak (less than 1% relative abundance) due to an isotopic cluster with its most abundant peak at *m/z* 815.1349 is attributed to [(GS)_2_Hg + H^+^] (+5.2 mDa); this species has been observed in previous reports of Hg(II) conjugated to GSH[8–11]. A 1:1 Hg(II):GSH conjugate is not observed immediately after mixing, but a curious isotopic cluster centered around *m/z* 544 having an isotopic pattern consistent with a single Hg(II) is observed. Only once has this species been reported in positive ion ESI mass spectra, and that was collected at low resolution; hence, the identity of the species remained ambiguous, although it was speculated that the species responsible “might be a Hg-GS cluster with two water molecules associated with it” [8]. However, as discussed below, high mass accuracy and MS/MS reveal another composition involving Hg(II). Interestingly, with time the 1:1 Hg(II):GSH conjugate appears, and after four days of incubation at room temperature, it becomes by far the dominant Hg(II)-containing species in the ESI-FTICR mass spectrum (Figure 1(b)); [(GS)_2_Hg + H^+^] is still present, but remains weak.

**Figure 2:**
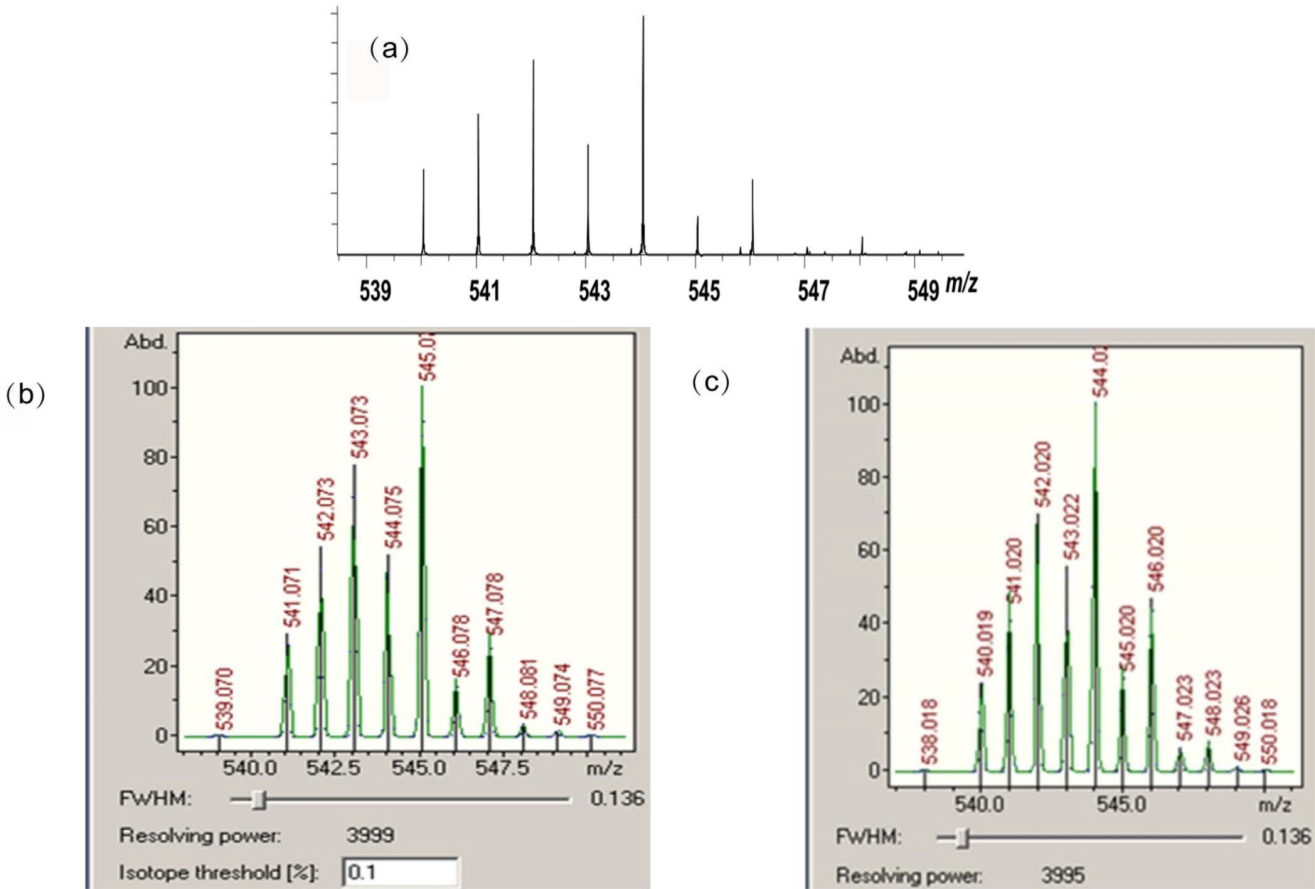
(a) Inset of ESI-FTICR mass spectrum of aqueous solutions of Hg(II) mixed with GSH after four days incubation at room temperature around m/z 544; (b) Simulated isotope distribution for [Hg(GSH) − H + 2H_2_O]^+^; (c) Simulated isotope distribution of [GSH + HgCl]^+^.

Intrigued by the fact that the species containing Hg(II) around *m/z* 544 decreases with time while the 1:1 Hg(II):GSH conjugate around *m/z* 508 increases with time, we compared the high mass accuracy for the *m/z* 544 conjugate and its isotopic distribution against that previously predicted [8]. Figure 2(a) represents an inset of the isotopic distribution measured around *m/z* 544 by ESI-FTICR, and bears a striking similarity to the isotopic distribution reported for this ion by Krupp *et al*. in Figure 2d of their paper [8], suggesting these are in fact due to the same ionic species. However, this experimentally-derived result does not match well with the theoretically-predicted isotopic distribution for the postulated [Hg(GSH) − H + 2H_2_O]^+^ (Figure 2(b)) [8]. Accurate mass measurement of the nominal *m/z* 544 peak by ESI-FTICR is 544.0263 Da, which further indicates [Hg(GSH) − H + 2H_2_O]^+^ cannot be the identity of this ionic species. However, this accurate mass measurement is in close agreement with another possible formula corresponding to [GSH + HgCl]^+^; a simulation of its theoretical isotope distribution is shown in Figure 2(c). The experimental ESI-FTICR data in Figure 2(a) agrees well not only with the theoretical isotopic distribution, but also with the predicted ionic mass (544.0217 Da) with only 4.6 mDa error.

**Figure 3:**
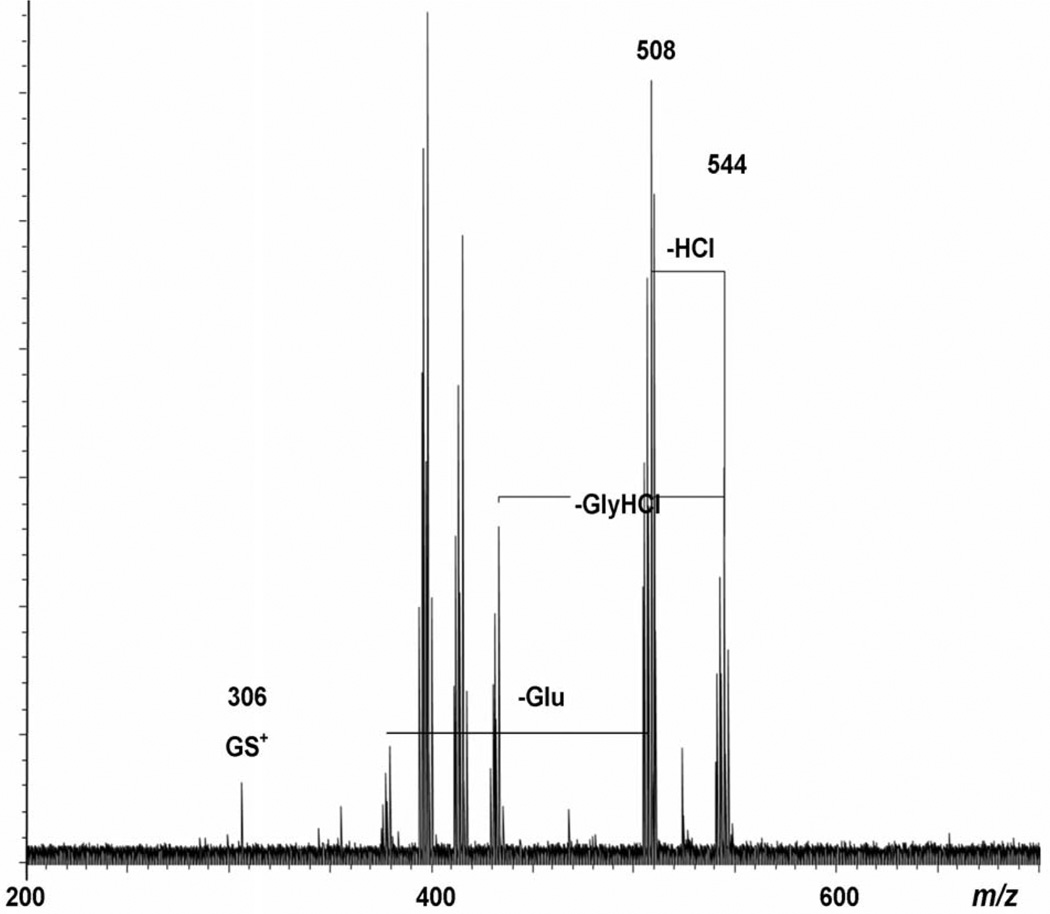
CID(12V) of the *m/z* 544 cluster generated by ESI-FTICR.

To validate that chlorine was indeed present in this species, we conducted MS/MS using CID. The entire isotopic cluster centered about *m/z* 544 was mass-selected in the source quadrupole of the SolariX FT-ICR and dissociated with argon gas in the collision cell before transport to the FT-ICR ion trap. CID experiments were conducted over the range 8 – 16 V, but the product ions generated only showed small differences in relative abundance and not in the actual product ions produced, so only the 12 V CID result is shown in Figure 3. CID confirms that chlorine is present in the formula for *m/z* 544; neutral loss of 35.9764 Da is observed, corresponding to loss of HCl. The neutral loss of 111.0079 Da is due to the loss of glycine hydrochloride from the C-terminus of GSH, which is followed by subsequent neutral losses of H_2_O and NH_3_, respectively. The peak at *m/z* 379.0032 Da is consistent with the loss of the N-terminal μ-glutamic acid residue [17,18] from the *m/z* 508 product ion, while *m/z* 306.0756 corresponds to GS^+^; these results completely support a previously reported dissociation mechanism of the 1:1 Hg(II):GSH conjugate [9].

It is curious to consider how an aqueous solution of mercuric nitrate would produce this intriguing intermediate species. It should be noted that commercial mercuric nitrate does contain low levels of chloride impurities. Thus, GSH may form the intermediate by the following reaction:

(1) $$GSH+{HgCl}_{2}\to {\left[GSH+HgCl\right]}^{+}+{Cl}^{-}$$

**Scheme 1:**
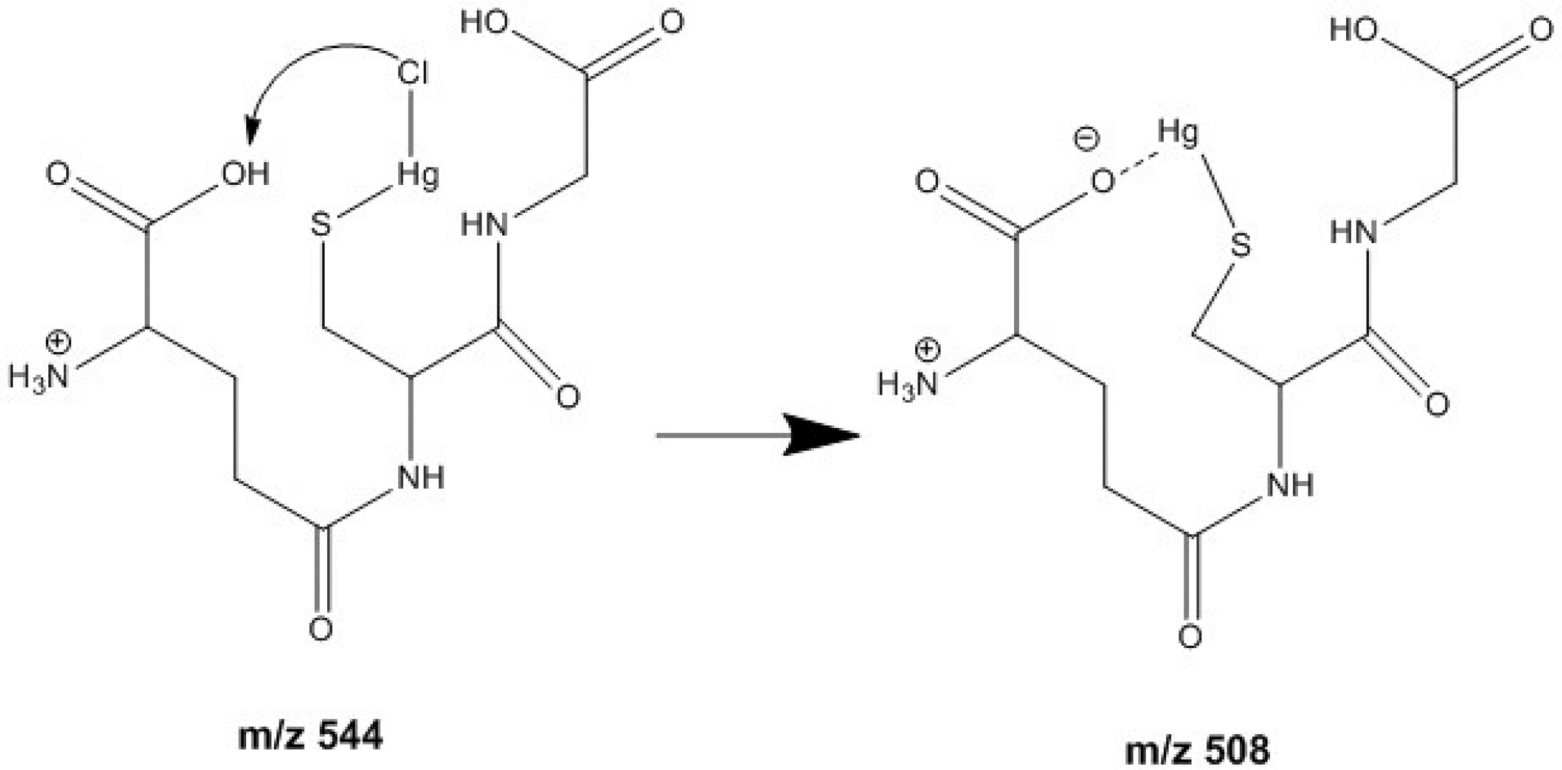
Fragmentation of [GSH + HgCl]^+^ intermediate.

In order to have such a composition with Hg(II) bound at the thiol, to maintain the overall mass of GSH, the N-terminus must be in its protonated form. If so, then the neutral loss of HCl can be explained as shown in Scheme 1. The product ion would then match the zwitterionic structure proposed for *m/z* 508 [9]. Additional support for this interpretation is derived from the fragmentation observed by CID, where the loss of the N-terminal and C-terminal amino acids from the conjugate occurs without the loss of the mercury atom, implying that it is indeed covalently bound to the central Cys residue.

## 4. Conclusion

Previous low-resolution ESI-MS of Hg(II) conjugates with GSH was unable to establish conclusively the identity of an isotopic cluster centered about *m/z* 544, although it was postulated that such an ion might be a 1:1 conjugate with two associated water molecules. Here, the high mass accuracy capability of FT-ICR coupled to an ESI source of Hg(II)-GSH mixtures establishes that the isotopic cluster is [GSH + HgCl]^+^; further confirmation is established through the observed isotopic ratios, consistent with a species containing single Hg and Cl atoms, and CID, which shows neutral loss of HCl upon collisional activation of the precursor ion. Based on kinetics studies, the conjugate apparently forms immediately upon mixing. However, the conjugate species [GSH + HgCl]^+^ observed is unstable, and eventually decays into [GSH − H + Hg]^+^, an isotopic cluster centered about *m/z* 508, which has been reported previously. Thus, the high mass accuracy capability of FT-ICR provides insight into the mechanism of formation of a prominent organomercury thiol.
